# Metagenomic next-generation sequencing of plasma cell-free DNA improves the early diagnosis of suspected infections

**DOI:** 10.1186/s12879-024-09043-3

**Published:** 2024-02-12

**Authors:** Hui Zhang, Ruobing Liang, Yunzhu Zhu, Lifen Hu, Han Xia, Jiabin Li, Ying Ye

**Affiliations:** 1 Department of Infectious Diseases, The First Affiliated Hospital of Anhui Medical University, Hefei, China; 2 Department of Scientific Affaires, Hugobiotech Co., Ltd, Beijing, China; 3 Institute of Bacterial Resistance, Anhui Medical University, Hefei, China; 4 Anhui Center for Surveillance of Bacterial Resistance, Hefei, China

**Keywords:** Plasma cell-free DNA, Metagenomic next-generation sequencing, Early diagnosis, Suspected infections, Febrile illness

## Abstract

**Background:**

Metagenomic next-generation sequencing (mNGS) could improve the diagnosed efficiency of pathogens in bloodstream infections or sepsis. Little is known about the clinical impact of mNGS test when used for the early diagnosis of suspected infections. Herein, our main objective was to assess the clinical efficacy of utilizing blood samples to perform mNGS for early diagnosis of suspected infections, as well as to evaluate its potential in guiding antimicrobial therapy decisions.

**Methods:**

In this study, 212 adult hospitalized patients who underwent blood mNGS test in the early stage of suspected infections were enrolled. Diagnostic efficacy of mNGS test and blood culture was compared, and the clinical impact of mNGS on clinical care was analyzed.

**Results:**

In our study, the total detection rate of blood mNGS was significantly higher than that of culture method (74.4% vs. 12.1%, *P* < 0.001) in the paired mNGS test and blood culture. Blood stream infection (107, 67.3%) comprised the largest component of all the diseases in our patients, and the detection rate of single blood sample subgroup was similar with that of multiple type of samples subgroup. Among the 187 patients complained with fever, there was no difference in the diagnostic efficacy of mNGS when blood specimens or additional other specimens were used in cases presenting only with fever. While, when patients had other symptoms except fever, the performance of mNGS was superior in cases with specimens of suspected infected sites and blood collected at the same time. Guided by mNGS results, therapeutic regimens for 70.3% cases (149/212) were changed, and the average hospitalized days were significantly shortened in cases with the earlier sampling time of admission.

**Conclusion:**

In this study, we emphasized the importance of blood mNGS in early infectious patients with mild and non-specific symptoms. Blood mNGS can be used as a supplement to conventional laboratory examination, and should be performed as soon as possible to guide clinicians to perform appropriate anti-infection treatment timely and effectively. Additionally, combining the contemporaneous samples from suspected infection sites could improve disease diagnosis and prognoses. Further research needs to be better validated in large-scale clinical trials to optimize diagnostic protocol, and the cost-utility analysis should be performed.

**Supplementary Information:**

The online version contains supplementary material available at 10.1186/s12879-024-09043-3.

## Introduction

In the outpatient and hospital settings, fever is a common presenting symptom and may be observed in up to 30–50% of all medical patients during their hospital stay [1]. It is usually regarded as a beneficial host immune response to infection [2] but often leads to a series of diagnostic examinations which significantly raise medical costs and increase the risk of invasive procedures [3]. In addition to infection being the main cause of fever, there are hundreds of other causes, including myocardial infarction, pulmonary embolism, deep vein thrombosis, cerebral infarction, hemorrhage, atelectasis, drug fever, and postoperative fever [4]. Among hospitalized patients, although presentation of fever in most cases reminds to perform at least one microbiological test, it is not a predictor of positive pathogenic results [5]. Despite this, empirically antibiotic therapy is commonly initiated as a result of this nonspecific sign, which may result in the inappropriate usage of antibiotics and an increase in antibiotic-resistant pathogens [4]. Therefore, early and accurate differentiation between infectious and non-infectious fever is very important.

Clinical laboratory plays an important role in infection diseases control and usually performs microscopic examination, culture, identification to detect pathogens [6]. However, the limited sensitivity and detection capacity of these methods shoulder some of the blame for the failure of detecting pathogens in a considerable number of cases [7]. Other molecular techniques such as 16 S rDNA sequencing [8], MALDI-TOF mass spectrometry [9] or real-time PCR [10, 11] could help clinicians diagnose diseases, but require several priori assumptions limiting their application. The limitation of these techniques makes a significant proportion of fever be undiagnosed [12]. Several biologic markers, such as serum procalcitonin (PCT), C-reactive protein (CRP), tumor necrosis factor-α (TNF-α), and interleukin-6 (IL-6), have been tested for their ability to distinguish between infectious and non-infectious fever [13]. While, none of these markers have been proven to be powerful enough to be used in clinical practice. Metagenomic next generation sequencing (mNGS) using cell-free DNA (cfDNA) has been proven to be a promising tool in detecting pathogens from body fluids with higher sensitivities (75–91%) and specificities (81–100%) [14]. A growing body of evidence suggested mNGS using plasma cfDNA could improve the diagnosed efficiency of pathogens in bloodstream infections or sepsis [15–17].

So far, the majority of mNGS research on blood samples has primarily focused on sepsis. Studies have concluded that blood mNGS testing is helpful for the etiological diagnosis of sepsis with higher sensitivities (75-90%) compared to blood culture, and with a shorter turnaround time [18–22]. It also holds promise for diagnosing fever of unknown origin, suggesting that mNGS could significantly reduce unnecessary antibiotic consumption [23]. It should be considered in conjunction with the application of traditional techniques [24], or as a first-line investigation using blood samples [25]. Additionally, several studies have focused on the diagnostic value of blood mNGS in detecting pathogens from patients with acute hematogenous osteomyelitis [26], intra-abdominal infections [27], infective endocarditis [28], febrile neutropenia [29], and transplantation [30], among others, indicating that blood mNGS can be a suitable test.

Zuo et al. demonstrated that in hospitalized patients with suspected sepsis, the mNGS test showed better performance for patients with mild symptoms, prior antibiotic use, and early stage of infection than blood culture [18]. However, little is known about the clinical impact of mNGS testing when used for the early diagnosis of suspected infections with mild and non-specific symptoms. Herein, 212 patients who received mNGS tests from blood samples were enrolled and clinical data was retrospectively analyzed to evaluate the clinical performance and applicability of mNGS in the present study.

## Materials and methods

### Trial design and patients recruitment

Adult hospitalized patients who underwent blood mNGS test in the early stage of suspected infections in the Department of Infectious Disease, the First Affiliated Hospital of Anhui Medical University between October 2020 and January 2022 were enrolled. Patients with incomplete medical records, drug fever, and solitary fever (presented only once fever) were excluded. Clinical data of all patients, including baseline demographic characteristics, chronic illnesses/disabilities, laboratory test results, clinical diagnosis, antibiotic administration and prognosis were collected.

### Samples collection and standard of microbiologic diagnostics

Blood specimens were collected after getting the consent of the patients and sent for mNGS test. Simultaneously, conventional microbiological methods, such as blood smear, culture, β-D-glucan/galactomannan (BDG/GM) tests, serologic tests, PCR, the T-SPOT.TB test were performed according to the clinical necessity. If the patients were suspected of having other site infections, specimens of suspected infected sites were also be sent for mNGS and the above-mentioned methods. Standard of clinical microbiologic diagnostics was based on the above conventional methods.

### Metagenomic next-generation sequencing

Whole blood from each patient was collected into cell-free DNA blood collection tubes (BCT) (Streck, Inc., Omaha, NE, USA) and transported to Hugobiotech Co., Ltd. (Beijing, China) to perform mNGS. Human cells in samples were removed by centrifugation at 1600×g for 10 min, followed by 16,000×g for 10 min at 4℃. Cell-free DNA (cfDNA) was extracted from the supernatant using QIAamp DNA Micro Kit (QIAGEN, Hilden, Germany) according to the instruction. The extracted cfDNA concentrations were measured by Qubit 4.0 (Thermo Fisher Scientific, MA, USA). And then, metagenomics libraries were constructed by QIAseq Ultralow Input Library Kit (QIAGEN, Hilden, Germany) according to its manual. The qualified library was sequenced on Nextseq 550 platform (Illumina, San Diego, USA) using high-output flow cell at 75 cycles of single end sequencing.

The sequencing data were analyzed for pathogens using the optimized SURPI + computational pipeline [31]. After filtering out adapter, low-quality, low-complexity, and shorter reads of < 35 bp (24), high-quality sequencing data were generated. Next, human reads were removed by mapping reads to human reference genome (GRCh38) using Bowtie2 v2.4.3 [32]. The remaining clean data was aligned to the microbial genome database (ftp://ftp.ncbi.nlm.nih.gov/genomes/) using Burrow-Wheeler Aligner software (v0.7.17) [33]. The reads number and reads per million mapped reads (RPM) of each detected pathogen was calculated. In parallel with the clinical samples, positive (synthesize fragments with known quantities) control and negative control (non- template control, NTC (sterile deionized water)) were also set for each batch of experiments using the same wet lab procedures from DNA extraction to end of sequencing and bioinformatics analysis. Samples that failed to achieve unique PC reads for any reason resulted in a one-time requeue. The NTC samples enabled estimation of the number of background read [34].

### Criteria for positive mNGS detection

1. For the detected bacteria (*Mycobacterium* excluded), fungi (*Cryptococcus* excluded), and parasites, the positive criteria for the mNGS result were set as follows: (1) genome coverage of the unique reads mapped to this microorganism ranked top10 of the same kind of microbes and the microorganism was not detected in the NTC; or (2) RPMsample/RPMNTC was > 10 (RPMNTC ≠ 0).
2. For viruses, *M. tuberculosis*, and *Cryptococcus*, a positive mNGS result was considered when it was not detected in NTC and at least 1 unique read was mapped to species or when RPMsample/RPMNTC was > 5 (RPMNTC ≠ 0) [35].

After the prior analysis, the mNGS results were assessed by three independent board-certified infectious disease physicians, and clinical criteria outlined in the Karius test [36]. “Causative pathogens” were defined according to whether the detected microbes were the commonly reported pathogens and/or the infections caused by the microbes were in accordance with clinical features of patients, or the detected organisms would be classified as non-pathogenic microbes [36, 37].

### Diagnostic assessment

Infectious or non-infectious diseases were diagnosed by the comprehensive combination of epidemiology, clinical characteristics, laboratory test results, imaging results, mNGS and conventional diagnostic results, and treatment response, and were evaluated by at least two experienced physicians. The sensitivity, specificity and total coincidence rate (TCR, including positive and negative agreement) of mNGS was evaluated based on final clinical diagnosis.

### Statistical analysis

Categorical variables were described in absolute numbers and in percentages. Continuous variables were calculated using medians ± standard deviations (SD), and abnormal distributions were described by medians and interquartile ranges (IQRs). McNemar test or Chi-square test were used to evaluate independent binomial variables, taking *P* < 0.05 as statistically significant threshold. Data analysis were performed using SPSS V25.0 statistics software.

## Results

### Patients and sample characteristics

A total of 212 participants were retrospectively enrolled with a median age of 54.5 years (IQR 41–67 years), of whom 129 (60.8%) were male. According to the final clinical diagnosis, 184 of 212 patients were classified to the group of infectious disease (ID), including 25 suspected infections in which infection was clinically diagnosed but with negative microbiology tests. Twenty-eight patients were categorized into the non-infectious disease (NID). All the baseline characteristics of 212 patients were shown in Table 1, and there was not any significant difference in baseline data between ID and NID groups, including gender, count of white blood cell (WBC), percentage of neutrophils, percentage of blood lymphocytes, C-reactive protein (CRP), and clinical symptoms, except age and procalcitonin (PCT). One blood specimen was sent for mNGS testing from each of 154 (72.6%) patients, while ≥ 2 specimens from different locations at the same time were sent for mNGS testing from each of the remaining 58 patients (27.4%), resulting in the inclusion of 295 total samples (Table 1).

**Table 1:** Clinical characteristics of the enrolled 212 patients

Demographics	Infectious Group (*n* = 186)	Non-infectious Group (*n* = 28)	P value
**Male: Female**	1.63:1 (114:70)	1.15:1 (15:13)	0.39
**Age (years)**			< 0.01
Median	55.5 (44–69)	43 (28.5–57.5)	
Average (SD)	55.2 ± 18.2	44.3 ± 17.8	
**Days from onset (days)**	15(10–22)	12.5(8.5–17.5)	0.07
**Clinical Symptoms, n (%)**			
Fever	160 (86.9%)	26 (92.8%)	0.37
Muscle or joint pain	19 (10.3%)	0 (0.0%)	0.15
Abdomen pain with diarrhea	13 (7.1%)	1 (3.5%)	0.08
Cough	12 (6.5%)	0 (0.0%)	0.37
Headaches	7 (3.8%)	1 (3.5%)	0.63
Vomiting	2 (1.1%)	0 (0.0%)	1.00
**Sample types of mNGS, N**			
Blood	197	28	
CSF	12		
Ascitic fluid	10	2	
Pleural effusion	8	1	
Urine	7		
Sputum	6		
Pus	5		
BALF	2		
bone marrow	2		
Drainage and Puncture fluid	12		
Puncture	2	1	
**Blood routine examination**			
Whole blood cell (×10^9^/L)	7.2 (5.1–11.1)	6.4 (4.5–10.2)	0.56
Neutrophils %	73.5 (60.4–84.2)	68.1 (59.4–77.2)	0.08
Lymphocytes %	17.6 (8.8–26.0)	18.45 (12.7–27.3)	0.56
**Immune status**			0.96
Normal	174	27	
Immunocompromised	10	1	
**Examination findings**			
C-reactive protein (mg/L), median (IQR)	62.3 (14.3-124.9)	40.0 (12.1–74.2)	0.34
Procalcitonin (µg/L), median (IQR)	0.18 (0.08–0.77)	0.12 (0.07–0.57)	0.02

### The performance of cfDNA mNGS compared with conventional methods

**Fig. 1:**
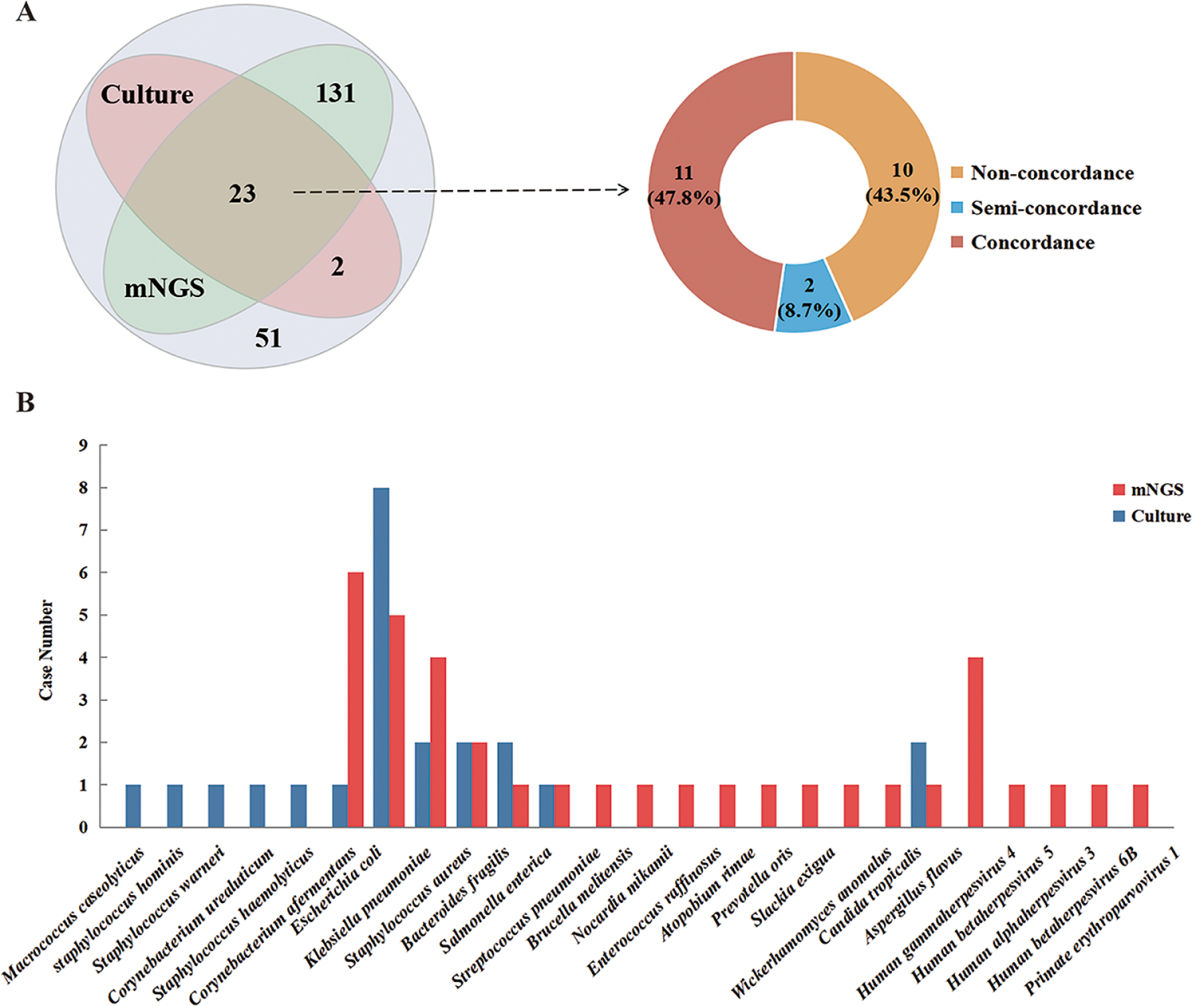
The comparison between blood mNGS and culture for potential pathogen detection. **(A)** The concordance between mNGS and culture for potential pathogen detection. **(B)** The pathogens detected by mNGS and culture in the both positive blood samples

In general, 89 microbes in total identified in 75.9% (224/295) specimens by mNGS, and bacteria (*n* = 65) were the most common organism, among which gram-negative bacteria accounted for 46.2% (30/65). Others included 10 fungi, 13 viruses, and *Cyclospora cayetanensis* (Supplementary Table 1). Microorganisms were detected in 165 blood samples, with a detection rate of 73.0% (165/226). A total of,15 pathogens were detected in 25 positive blood cultures (12.1%, 25/207). *E. coli* (*n* = 8) was the most common bacteria species, and others identified by culture were *Staphylococcus spp.* (*n* = 6), *Salmonella enterica* (*n* = 2), and *Klebsiella pneumoniae* (*n* = 2) and so on (Fig. 1). The two tests (culture and mNGS) were both positive in 23 of 207 specimens and were both negative in 51 specimens (Fig. 1A). Only two samples had only culture-positive results. 131 cases were mNGS test positive and culture negative. Rare and intracellular pathogens, such as *Brucella melitensis* (*n* = 2) and *Mycobacterium tuberculosis* complex (*n* = 7) were detected solely by mNGS. Overall, in the pairs of mNGS and culture, the total detection rate of mNGS was 74.4% (154/207), which was significantly higher than that of culture method (12.1%, 25/207; *P* < 0.001).

**Fig. 2:**
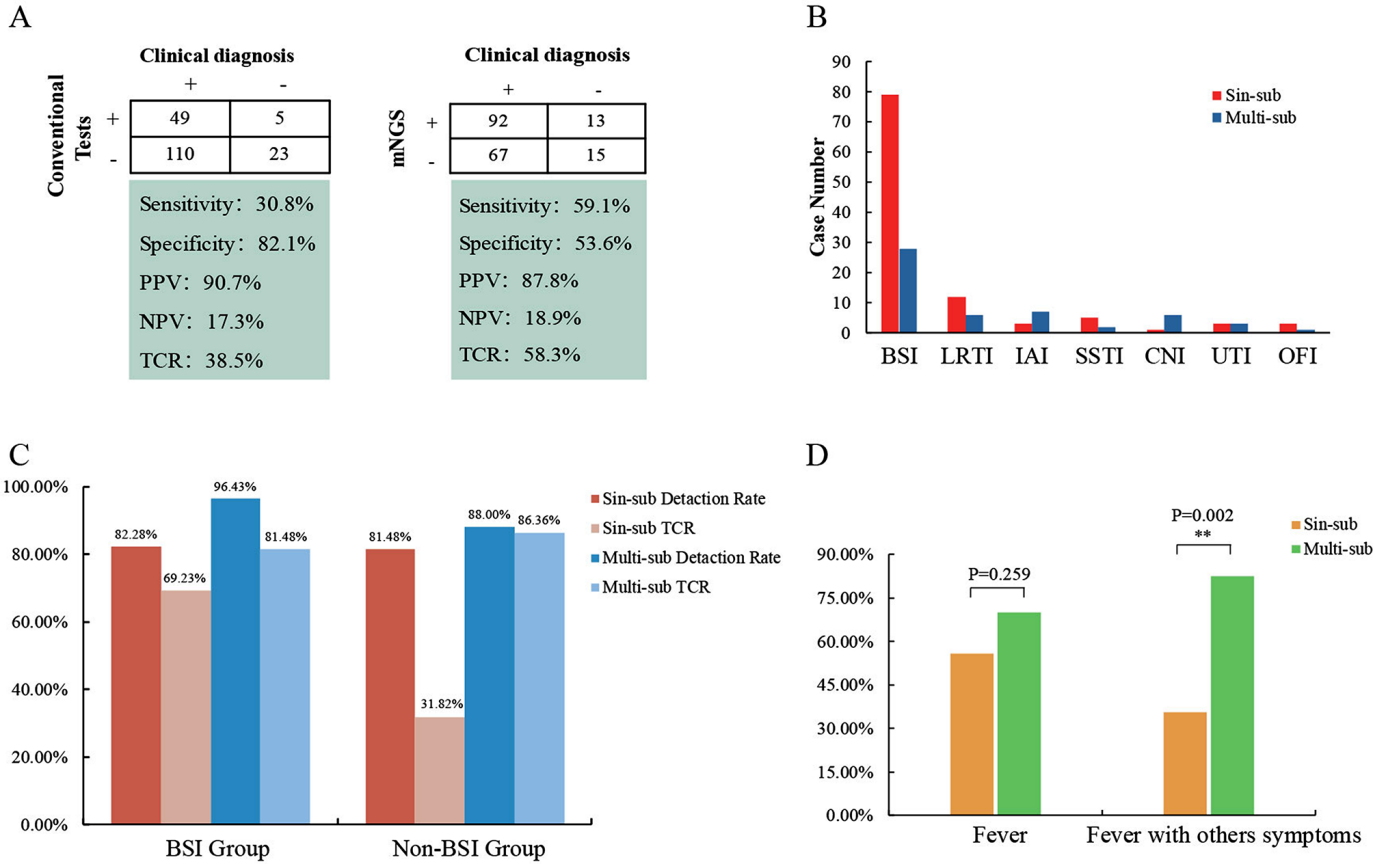
The diagnostic efficiency and performance of mNGS in identifying infectious and non-infectious disease. **(A)** The diagnostic efficiency by mNGS and conventional methods based on the clinical diagnosis. **(B)** Infection types of the enrolled cases based on final clinical diagnosis. **(C)** The comparison of detection rate and total coincidence rate (TCR) between BSI and non-BSI group. **(D)** The performance of mNGS in identifying infectious and non-infectious fever. *Abbreviations*: PPV, positive predictive value; NPV, negative predictive value; TCR, total coincidence rate; BSI, bloodstream infection; LRTI, lower respiratory tract infection; IAI, intra-abdominal infection; SSTI, skin or soft-tissue infection; CNSI, central nervous system infection; UTI, urinary tract infection; sin-sub, single sample subgroup; multi-sub, multiple samples subgroup

In addition, T-SPOT was performed in 153 patients and the results showed 36 patients had suspected TB infections. Other conventional methods, including GM tests, serologic tests, PCR, identified 42 positives, including 23 viral positives. Combined above conventional tests, the positive rate of detection was 43.4% (92/212). Finally, 187 patients got a definite diagnosis and the diagnostic information of the remain 25 cases was unclear. As expected, we found that mNGS showed approximately 28.3% higher sensitivity compared with conventional tests (59.1% vs. 30.8%; *P* < 0.001), although the specificity of mNGS testing was lower than that of conventional tests (53.6% vs. 82.1%; *P* = 0.04). Besides, 58.3% mNGS results were consistent with clinical diagnosis, which was superior than that of conventional methods (38.5%, 74/187) (Fig. 2A). These data demonstrated that cfDNA mNGS was superior in the early diagnosis of suspected infections than conventional methods.

### The diagnostic efficiency of mNGS in different infection types

In our study, blood stream infection (BSI) (107, 67.3%) comprised the largest component of all the diseases (Fig. 2B). Lower respiratory tract infections (LRTIs) were most common [18/52 (34.6%)] in the non-BSI group [52/159 (32.7%)], followed by intra-abdominal infections (IAIs) [10/52 (19.2%)] and skin or soft-tissue infections (SSTIs) [7/52 (13.4%)]. In the BSI group, 79 cases were only performed blood mNGS (single sample subgroup, sin-sub), and two more types of specimens (multiple samples subgroup, multi-sub) were collected in 28 cases. The detection rate and positive coincidence rate in multi-sub were slightly higher but with no significant difference (82.3% vs. 96.4%; 69.2% vs. 81.5%). Additionally, in the non-BSI group, 25 cases were assigned to multi-sub and the positive coincidence rate significantly increased, compared with sin-sub (86.4% vs. 31.8%, *P* < 0.001) (Fig. 2C). When we focused on the muti-sub in both BSI and non-BSI group, we found the same pathogen was detected in different samples from the same individual in nearly half of the cases, indicating that although patients presented with mild and non-specific symptoms such as malaise, fever and dry cough, bloodstream infection has crept up in the patients’ body. (Supplementary Table 2).

### The performance of mNGS in identifying infectious and non-infectious fever

We further compared the ability of mNGS tests to distinguish ID from NID and found the PPV and NPV of diagnosing ID by mNGS were calculated to be 87.8% and 18.9%, respectively (Fig. 2A). The 187 patients complained with fever when they visit to hospital, of whom 46 presented extra symptoms such as muscle or joint pain, cough, diarrhea etc. Among sin-sub and multi-sub cases, there was no difference in the diagnostic efficacy of mNGS in cases presenting only with fever. While, when patients had other symptoms except fever, the performance of mNGS was superior in cases with specimens of suspected infected sites and blood collected at the same time (Fig. 2D).

**Fig. 3:**
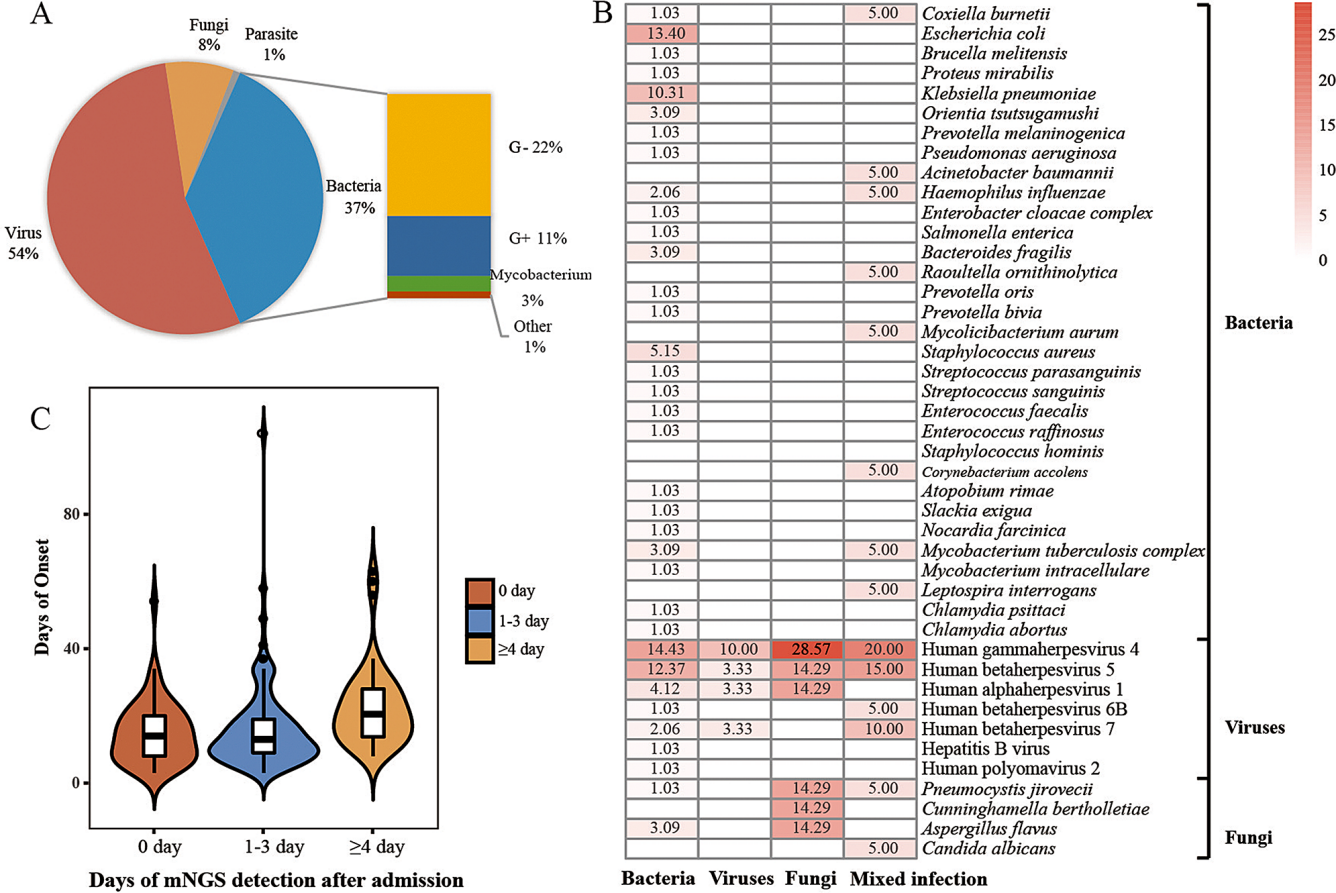
Pathogen profiles presented by mNGS based on the final clinical diagnosis and the application of mNGS in assisting clinical care. **(A)** Distribution of potential pathogens identified by mNGS in blood samples. **(B)** The pathogens profiles presented by plasma cfDNA mNGS based on the final diagnosis. The number presented in the box was presented as the ratio of the case number of the pathogen relative to the total number of detected pathogens per patient in bacterial, fungal, viral or mixed infections. **(C)** The relationship between average hospitalized days and sampling time

Sixty kinds of pathogens were detected in blood, including 40 bacteria, 11 viruses, 8 fungi and one parasite. Viruses were detected in more than half of blood specimens, followed by bacteria (Fig. 3A). Combined with clinical manifestations, we found bacterial bloodstream infections were the most common infections among the febrile illness patients. We also detected co-infections of > 1 pathogen in this study (*n* = 13), with bacterial and viral co-infections being the most common type (Supplementary Table 3). The pathogen profiles showed the main causative pathogens identified in bacterial infections were *E. coli* with contributions 13.4% (Fig. 3B). *Pneumocystis jirovecii*, *Aspergillus flavus* and *Cunninghamella bertholletiae* were the causative pathogens in fungal infections with contributions 14.29%, respectively. The above dominant pathogens were not identified in viral and mixed infections, except *P. jirovecii.* Human gammaherpesvirus 4 (EBV) contributed the most (10% ~ 28.57%) in all types of infection, followed by Human betaherpesvirus 5 (CMV) with contributions from 3.33 to 15%. While, the clinical irrelevant EBV, CMV, and other viruses were identified with a low RPM (1-148) in bacterial and fungal infections.

### The application of mNGS in assisting clinical care

mNGS could play an important role in assisting clinical care and therapy. Totally, anti-tuberculosis treatment was performed in 15 patients, with three patients owned positive results of mNGS and T-SPOT, and 4 patients with positive mGNS detection but negative T-SPOT. Among the 11 patients with positive GM test, fungi were detected by mNGS in 5 patients and two patients received anti-fungal treatment. Four other patients with confirmed fungal infections had positive mNGS tests but negative GM tests. Although viruses were detected in more than half of blood specimens, only 15 patients with positive virus results received anti-viral treatment. Guided by mNGS results, therapeutic regimens for 70.3% cases (149/212) were changed (Table 2). Fourteen patients completely or partially stopped unnecessary medication and 10 patients were treated with additional drugs. Complete or partial changes in types of drugs were carried out on 80 and 24 cases, respectively, 37 of whom received changed treatment regimens only according to mNGS results. Ultimately, most (82.5%, 175/212) of patients recovered and discharged, and 36 patients demonstrated improvement. Only one 80-year-old patient died due to complications after pulmonary infection. Additionally, specimens for mNGS tests were collected from 56, 104, 52 patients on the 0, 1 ~ 3 and ≥ 4 days of admission, respectively, and the average hospitalized days were significantly shortened in the former two groups (15.1 days for sampling time of day 0, 16.2 days for sampling time of day 1 ~ 3, and 22.5 days for sampling time ≥ day4, Fig. 3C), which suggested performing mNGS test as soon as possible can shorten hospitalized days and reduce hospitalization costs.

**Table 2:** Impact of mNGS on antimicrobial treatment on suspected infections

Modifications	Patients (*n* = 212)
Remove unnecessary agents	14 (6.60%)
Add agents	10 (4.72%)
Change completely	80 (37.73%)
Change agents partially	24 (11.32%)
No change	63 (29.72)

## Discussion

In this study, we found the detection rate of blood mNGS was significantly higher than that of culture method. Although patients presented with mild and non-specific symptoms such as malaise, fever and dry cough, bloodstream infection has crept up in host body, where blood mNGS can be a promising role. If people just complained with fever, clinicians can perform blood mNGS firstly to identity possible infections. Guided by mNGS results, therapeutic regimens could be changed positively, and the average hospitalized days could be shortened.

Progress in genome sequencing provides hope for overcoming diagnostic challenges, of which mNGS has been proven to the utility of detecting nearly all known pathogens from clinical samples [38–40]. In our study, potential pathogens were detected by conventional methods and cfDNA mNGS at the same time, and the latter had a high sensitivity (59.1%), approximately 28% higher than that of conventional methods. Meanwhile, our results confirmed that cfDNA mNGS was superior in pathogen detection with higher TCR (58.3%). Although culture is the most common approach for most cases of infection, the limitations were partially attributable to technical shortfalls in blood culture acquisition as well as to local foci, uncultivable or fastidious organisms, or very low rates of viable microorganisms in the specimens [14]. Compared with blood culture, plasma cfDNA mNGS can detect a much wider pathogen spectrum, of which more than two microbes were detected in 76 cases in our study. Another limitation of blood culture is that certain pathogens require longer growth time on culture media [41], for example, *M. tuberculosis* generally takes 2–4 weeks or even longer to grow, and, and *Brucella* usually takes more than one month [23]. In contrast, mNGS can obtain results within 24 h after sampling, greatly reducing the detection time. In our culture negative specimens, mainly detected microorganisms were difficult to culture or have a long culture time, such as *B. melitensis*, *Chlamydia psittaci*, and *M. tuberculosis*, further providing mNGS is a faster and more effective method for detecting rare and uncommon pathogens.

As a non-invasive diagnosis, it can diagnose possible infections by capturing and identifying highly fragmented cfDNA in the blood [42], which is a double-edged sword in terms of diagnostic performance. The increased false positives reduced the specificity of blood mNGS. Several reasons could result in the false-positive results such as the abnormal host conditions (overgrowth of intestinal commensal organisms, increased permeability of the intestinal mucosal barrier, or compromised immune defence), contamination during sample collection, contamination from commercial kits, analysis errors including index hopping, nonspecific alignment with human sequences or similarity species [43]. In our study, the false positive was likely caused by increased mucosal barrier permeability or decreased host immune defense, especially in cancer/ autoimmune/ transplant patients [44]. As microbial cfDNAs are very short and their concentrations vary significantly, it poses significant challenges to the clinical interpretation of the results. In our study, the false-positives presented low specific reads (1 to 394), suggesting that the pathogenicity of microbes detected by blood mNGS with low load should be evaluated comprehensively combining clinical symptoms and other test results. In the future, clinicians also should construct a negative-control microorganism database to filter out noise signals and reduce false-positive results. Another unexpected finding was that *Macrococcus caseolyticus*, two *Corynebacterium spp.*, and three *Staphylococcus spp.* only resulted in blood culture, and were failed to detected by blood mNGS. Gram-positive bacteria, mycobacteria, and fungi, which own the rigid cell walls, require bead beating or enzymatic treatment for adequate recovery of DNA [45], however this could increase the host-background of human DNA [9]. In our study, mNGS tests were performed using cfDNA without host depletion. Hence, we attributed the lower sensitivity of the above bacteria detection by the low concentration of the species due to extraction method. In the future, the extraction method of pathogen DNA still needs to be further optimized, and deepening the sequencing coverage of samples can be another method.

Previously, O’ Grady commented that nucleic acids of pathogens could be detected from plasma even if the infection was confined to a specific anatomic location [36], and a single center study reported that 34 invasive techniques were avoided due to mNGS results [46]. In our study, pathogens in seven cases only detected by plasma cfDNA mNGS in the non-BSI group indicated infection in other parts of the body, including LRI, IAI, and UTI. The cfDNA of invasive pathogens might opportunistically enter the blood when the tissue mucosa was damaged by local infection or physical damage, resulting in bacteraemia or viraemia [44, 47]. However, the load of pathogen cfDNA in loci sites was significantly higher than that in blood in matched samples [14]. In our study, compared with focal sites, bacterial DNA presented in the blood at lower load in the non-BSI group, and the TCR was higher in multi-sub, indicating that in order to make definite diagnosis as early as possible and improve prognosis, specific locations and blood specimens should be collected at the same time for testing as soon as possible.

In our study, fever was the frequent presenting complaint from patients in the outpatient setting, and it is one of the most common reasons for seeking health care globally [48]. Possible causes of febrile illness include a wide spectrum of pathogens such as bacterial bloodstream infections, zoonosis, protozoal infections, fungal infections and viral infections [49]. Bacterial bloodstream infections were the most infections in our study, including *E. coli*, *K. pneumoniae*, *Staphylococcus aureus*, and other Gram-negative organisms, consistent with the previous studies [5, 12]. However, *Salmonella enterica*, which was the most common bacterial pathogen in south and southeast Asia [50], was only detected in one case in our study. Due to different treatments among diseases, it is important to recognize the cause of infection, even if most patients only have mild symptoms. Additionally, zoonotic infections including *Coxiella burnetii*, *Orientia tsutsugamushi*, *Chlamydia abortus*, *C. psittaci*, *B. melitensis*, *Leptospira interrogans*, Hantaan Virus, and severe fever with thrombocytopenia syndrome virus, were identified in the 9.4% (15/159) of infectious cases in our study. The close association between people, livestock, and wildlife in city and countryside of Anhui province is an important driver of the high prevalence of zoonosis.

In our study, we detected 10 fungal species in 33 specimens from 24 patients, with 8 species found in the blood. At present, fungal infections are particularly difficult to diagnose by traditional means, and only 6 patients in our study were diagnosed with fungal infection. As shown in Supplementary Table 1, *Aspergillus* spp. was detected in nearly 40% of the fungal positive specimens. Previous study demonstrated that depending on the underlying immune status of the host, *Aspergillus* species can cause a wide spectrum of diseases in humans, including chronic pulmonary aspergillosis caused by colonization and proliferation of the fungus [51, 52], allergic bronchopulmonary aspergillosis in atopic patients [53], and invasive pulmonary aspergillosis in the immunocompromised patients [54]. Until now, only two types of circulating molecules, cell wall polysaccharide-based antigens and fungal DNA can be identified to date in biological fluids of invasive aspergillosis [55]. However, in contrast to viruses and bacteria, *Aspergillus* cells do not circulate, and the origin and source of the DNA remains undefined. In our study, the *Aspergillus* detected in blood samples with low specific reads (1–35). Due to our inability to determine whether the DNA detected was associated with active fungal growth or originated from the degradation of the fungus,, we could not rely on the blood mNGS results for the diagnosis of fungal infection. There were two patients diagnosed with *A. flavus* due to the fungus was identified both in blood and ascitic fluid, and their galactomannan serum tests were positive. Hence, a combination of cfDNA sequencing and GM/galactomannan serum tests could be an efficient diagnostic strategy to reduce unnecessary antifungal therapy.

Viruses were detected by cfDNA mNGS in over half (119/226, 52.6%) blood samples in our study. While, according to clinical characteristics, most viruses were classified as clinical irrelevant microbes. There is still no uniform standard to determine whether the viruses detected in blood were pathogenic or virus-carrying [42]. As reported that some viruses may shed from other body sites rather than reactivated from the blood [56, 57]. In practice, the clinical significance of the virus needs to be determined based on the patient’s medical history and clinical symptoms. In our study, we preformed PCR among highly suspected viral infectious patients to conform the reliability of viruses detected by mNGS. Finally, only six patients were diagnosed with viral infection, three of whom showed PCR positives, and another patient was diagnosed with Hantaan virus by PCR test. Other patients only had positive mNGS results and finally made diagnosis of viral infections according to the antiviral treatment and good prognosis. EBV is one of the most common viruses in humans [58], and around 95% of the human population is infected with EBV [59]. Therefore, the presence with a low RPM (1-148) of EBV in blood samples is not surprising. Infection with CMV is also common throughout the globe, which accounts for 60% of adults in developed countries and more than 90% in developing countries. The virus can establish lifelong latent infections of the host, and immunosuppression is a key trigger of CMV reactivation [60]. Among our CMV patients, most of them had medical history, including HIV, systemic lupus erythematosus, diabetes and other immunosuppressive treatment. Whereas, a multi-center retrospective cohort study showed the clinical impact in mNGS positive cases only involved bacteria/ fungi but not DNA viruses [61]. In our study, as majority of cases were bacterial infections, we could not evaluate the clinical impact in bacterial/fungal/viral infections accurately.

In the end, there were still some deficiencies in our study. Both culture and mNGS lack specimen-specific standards to identify whether detected pathogenic microorganisms are derived from infection, colonization, or contamination. The gold standard for the diagnosis of infectious diseases in our study was based on comprehensive consideration, including epidemiology, clinical manifestations, laboratory test results, imaging results, mNGS and conventional diagnostic results, and outcomes after anti-infective treatments. Confirmatory tests, such as bacterial 16 S rDNA PCR or fungal 28 S rDNA-ITS PCR, were not performed on the samples. In addition, our cfDNA mNGS lacks of detection of RNA virus pathogens, and we performed serological tests or qPCR to detect suspected RNA viruses, which could result in the omission of some rare viruses. When single-stranded DNA viruses exist in the body in single-stranded form, mNGS would fail to detect them, resulting in false negative results. Finally, the research was limited by the single-center study and the relatively small sample size. The results need further validation in larger-scale clinical trials with comprehensive consideration and rigorous experimental design.

## Conclusion

In this study, we emphasized the importance of blood mNGS in early infectious patients with mild and non-specific symptoms. Blood mNGS can be used as a supplement to conventional laboratory examination, and should be performed as soon as possible to guide clinicians to perform appropriate anti-infection treatment timely and effectively. Additionally, combining the contemporaneous samples from suspected infection sites could improve the infections diagnosis and prognoses. Further research needs to be better validated in large-scale clinical trials to optimize diagnostic protocol, and the cost-utility analysis should be performed.

### Electronic supplementary material

Below is the link to the electronic supplementary material.

**Supplementary Material:** Supplementary Table 1~3

## Data Availability

The datasets used and/or analyzed during the current study are available at National Genomics Data Center (http://ngdc.cncb.ac.cn), reference number PRJCA013636.

## Abbreviations

mNGS: Metagenomic next-generation sequencing
PCR: polymerase chain reaction
cfDNA: cell-free DNA
RPM: reads per million
NTC: non- template control
TCR: total coincidence rate
ID: infectious disease
NID: non-infectious disease
BSI: blood stream infection
LRTIs: lower respiratory tract infections
IAIs: intra-abdominal infections
SSTIs: soft-tissue infections
UTI: urinary tract infection
EBV: Human gammaherpesvirus 4
CMV: Human betaherpesvirus 5
